# TIMolol Nasal Spray as a Treatment for Epistaxis in Hereditary Hemorrhagic Telangiectasia (TIM-HHT)—A Prospective, Randomized, Double-Blind, Controlled, Cross-Over Trial

**DOI:** 10.3390/pharmaceutics14112335

**Published:** 2022-10-29

**Authors:** Kornelia E. C. Andorfer, Florian Zeman, Michael Koller, Judith Zeller, René Fischer, Caroline T. Seebauer, Veronika Vielsmeier, Christopher Bohr, Thomas S. Kühnel

**Affiliations:** 1 Department of Otorhinolaryngology, University Hospital Regensburg, Franz-Josef-Strauss-Allee 11, D-93053 Regensburg, Germany; 2 Center for Clinical Studies, University Hospital Regensburg, Franz-Josef-Strauss-Allee 11, D-93053 Regensburg, Germany; 3 Department of Internal Medicine II, University Hospital Regensburg, Franz-Josef-Strauss-Allee 11, D-93053 Regensburg, Germany

**Keywords:** timolol, nasal spray, HHT, hereditary hemorrhagic telangiectasia, Morbus Osler, orphan disease

## Abstract

To date, there is no approved local therapeutic agent for the treatment of epistaxis due to hereditary hemorrhagic telangiectasia (HHT). Several case reports suggest the topical use of timolol. This monocentric, prospective, randomized, placebo-controlled, double-blinded, cross-over study investigated whether the effectiveness of the standard treatment with a pulsed diode laser can be increased by also using timolol nasal spray. The primary outcome was severity of epistaxis after three months, while the main secondary outcome was severity of epistaxis and subjective satisfaction after one month. Twenty patients were allocated and treated, of which 18 patients completed both 3-month treatment sequences. Timolol was well tolerated by all patients. Epistaxis Severity Score after three months, the primary outcome measure, showed a beneficial, but statistically nonsignificant (*p* = 0.084), effect of additional timolol application. Epistaxis Severity Score (*p* = 0.010) and patients’ satisfaction with their nosebleeds after one month (*p* = 0.050) showed statistically significant benefits. This placebo-controlled, randomized trial provides some evidence that timolol nasal spray positively impacts epistaxis severity and subjective satisfaction in HHT patients when additively applied to standard laser therapy after one month. However, the effect of timolol was observed to diminish over time. Trials with larger sample sizes are warranted to confirm these findings.

## 1. Introduction

Recurrent epistaxis is considered the most common clinical manifestation in hereditary hemorrhagic telangiectasia (HHT). It affects over 90% of HHT patients and has great impact on their quality of life [1,2]. Despite growing knowledge about the pathophysiology of the disease, there is no definite cure for HHT and treatment remains symptomatic. Treatment modalities to control nasal bleeding include nasal mucosal care, minor and major surgeries, as well as drug therapy, for example local and systemic treatment with tranexamic acid and bevacizumab. Ablative therapies for nasal telangiectasias, such as diode lasers or ND:YAG lasers, are recommended in patients have failed to respond to moisturizing topical therapies [3]. Because of the chronic nature of the disease, treatment of nasal manifestations has to be repeated frequently. However, because laser therapy is not always sufficient, other therapy methods, particularly those simple, durable, and lacking in side effects, are required.

In 2012, the first report on the successful application of the nonselective beta-adrenergic receptor antagonist timolol to treat nosebleeds in HHT patients was published [4]. Though not yet fully understood, several explanations on the mechanism of action of this drug have been proposed: First, this medication might induce vasoconstriction of capillaries, thus restricting blood flow. Second, it might inhibit the release of vascular endothelial growth factor (VEGF) and the differentiation of hemangioma progenitor/stem cells (HemSCs). Finally, apoptosis might be induced in rapidly growing vascular cells within the arteriovenous convolutes [5,6,7]. Three case reports published on the use of timolol in the treatment of HHT patients generally indicate that the topical application of timolol decreases nosebleeds in HHT patients [4,8,9]. Thereupon, we planned to investigate this effect in a placebo controlled trial. Moreover, we considered that the effect of timolol may be increased if the HHT foci are previously obliterated by laser in advance. Thus, the impact on growth inhibition could be more pronounced.

Therefore, we hypothesized that timolol nasal spray used for three months in addition to laser therapy will further decrease epistaxis in HHT patients compared to placebo nasal spray.

## 2. Materials and Methods

### 2.1. Study Design, Approval and Registration

The study was a monocentric, prospective, randomized, placebo-controlled, double-blinded, cross-over (AB/BA design) trial. The Ethics Committee of the University of Regensburg (Approval number: 18-1324-111), as well as the German Federal Institute for Drugs and Medical Devices (Approval number: 61-3910-40433637) approved the study proposal prior to data collection. Each patient gave written informed consent for participation and publication of results. The study was registered at DRKS (https://www.drks.de, accessed on 10 March 2020) main-ID: DRKS00020994). The Center of Orphan Diseases (FSER e.V./Förderverein für seltene Erkrankungen, Regensburg, Germany) of the University Hospital Regensburg and the German HHT foundation (Morbus Osler Selbsthilfe e.V., Berlin, Germany)supported the study financially. A detailed description of the study protocol has been published elsewhere [10]. For the sake of completeness, this section again summarizes the most important aspects of the course of the study.

### 2.2. Recruitment of Patients

HHT patients of both sexes and above 18 years were recruited at the outpatient clinic of the Department of Otorhinolaryngology at the University Hospital Regensburg. Patients who were interested in participating in the trial were contacted by telephone and an appointment for the enrollment visit was planned at the outpatient clinic of the Ear-Nose and Throat (ENT) Department of the University Hospital Regensburg.

To be eligible for enrollment, the patients had to meet at least 3 of 4 Curaçao criteria for HHT and had to be treated with a pulsed diode laser with a wavelength of 445 nm (Wolf TruBlue™ laser system/ARC Laser GmbH, D-90411 Nuremberg, Germany) in the area of the nasal mucosa at least twice. The incidence of their nosebleeds must have been at least once a week, with one of them lasting at least 5 min.

Exclusion criteria according to patient reports and review of medical documentation available at the time of study inclusion were: pregnancy, bronchial or chronic obstructive pulmonary disease, bradycardia, hypotension, severe peripheral circulatory disorders, cardiac failure, cardiac contraindications to beta blockers, patients with diabetes, hyperthyroidism, pheochromocytoma, muscle weakness or myasthenia gravis, severe anaphylactic reactions to various allergens, patients performing or planning desensitization, patients with hypersensitivity to the IMP or placebo in past medical history, patients with concomitant ingestion of oral medication interfering with beta blocker treatment (e.g., CYP2D6 inhibitors, calcium channel blockers) or concomitant medication with anticoagulants or beta blockers (systemically or ophthalmologically). Termination of existing iron therapy with iron tablets or infusions or the start of iron therapy with iron tablets or infusions within one month before study enrolment until conclusion of the study was prohibited, as was endonasal surgery under general anesthesia within the last three months before and during participation [10].

### 2.3. Randomization and Blinding

At visit 1, each patient was registered and given a patient identification number. A total of 20 patients were randomized, with each patient receiving both therapies and only the order of the therapies being randomized. For this purpose, the Center for Clinical Studies (ZKS) created a randomization list using SAS 9.4 software. To ensure equally distributed sequences between the patients, a block-wise randomization using a block size of *n* = 10 was used. The Department of Pharmacy of the University Hospital of Erlangen produced a double-blinded sequence of nasal sprays according to the randomization list. This procedure guaranteed allocation concealment throughout the study.

### 2.4. Methodology

Laser therapy of the arteriovenous shunts of the nasal mucosa under local anesthesia is the standard treatment for HHT patients at the University Hospital Regensburg [11,12]. The main objective of this monocentric, prospective, randomized, placebo-controlled, double-blinded, cross-over (AB/BA design) study was to evaluate the efficacy of the additional use of timolol nasal spray twice a day (daily dosage 1 mg) for three months compared to standard laser treatment (pulsed diode laser with a wavelength of 445 nm by Wolf TruBlue™ laser system/ARC Laser GmbH, D-90411 Nuremberg, Germany) alone. At the end of the trial, each patient had received both timolol and placebo treatment, as the study had a cross-over design. Patients were randomly assigned to either treatment arm (timolol/placebo or placebo/timolol).

Because of the short half-life of timolol nasal spray of two to five hours and the necessary repetition of the standard therapy every 3 months, we considered a wash-out period of twelve hours to be sufficient [13]. All results were adjusted for carry-over and period effects.

**Figure 1:**
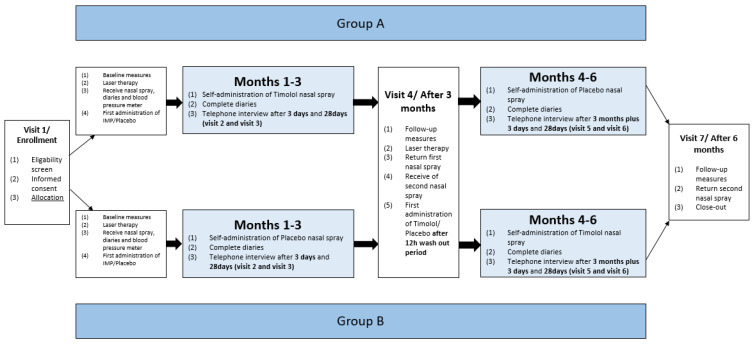
Flowchart of the study schedule.

Following the intake visit (visit 1), the patients had 6 consecutive follow-up visits. An overview of the course of the study is shown in Figure 1. A detailed flow schedule of all visits has been published elsewhere [10].

### 2.5. Sample Size

Sample size calculation for the trial resulted in a total sample size of *n* = 19 patients to achieve a power of 80% for the analysis of the primary endpoint. A detailed description of the sample size calculation can be found in the published study protocol [10].

### 2.6. Study Medication

The Investigational Medicinal Product (IMP) was timolol 0.5% 10 mL nasal spray (0.05 mL per burst = 0.25 mg timolol per burst). The placebo was the carrier solution of timolol nasal spray, which is benzalkonium chloride and phosphate buffer 10 mL nasal spray (0.05 mL per burst). The production of both, the IMP and the placebo, as well as the blinding was carried out by the Department of Pharmacy of the University Hospital of Erlangen.

### 2.7. Study Endpoints

The primary endpoint of this clinical trial was the Epistaxis Severity Score (ESS) three months after therapy. The ESS is an established and validated questionnaire for measuring the severity of epistaxis in HHT patients. The ESS ranges from 0 to 10 (mild to severe disease) and the minimal important difference (MID) of this value is 0.71 [14,15]. The questions refer to the symptoms experienced over the course of the last three months.

Secondary endpoints were blood values (hemoglobin, ferritin, and transferrin), quality of life as assessed using the EQ-5D-3L and the Morbus Osler symptom- specific questionnaire (SFB) assessed at the beginning of the study and after each treatment session, as well as the ESS one month after the beginning of each treatment session [16,17]. The EQ-5D-3L is a standardized questionnaire for simple, generic measure of health status. The SFB contains a total of 18 questions items, 13 of which are closed and 5 of which are open, on relevant symptom complexes of HHT, such as type and extent of symptoms, impairment of quality of life, activities and life planning, etc. The contents of the SFB can be found on the Internet at www.thieme-connect.de (accessed on 9 February 2006). Knowing that these questionnaires may not be sensitive enough to detect changes due to patients’ nosebleeds, they were the best available questionnaires at the time of study design. At this point, it must be emphasized that when the ESS was queried after one month, the symptoms of the last month were queried, not those of the last three months. Furthermore, subjective satisfaction of HHT patients regarding their epistaxis was evaluated before, during, and after taking the IMP by means of the single item worded “subjective satisfaction with my nose bleeding”, to be reported on a Numerical Rating Scale (NRS) ranging from 0 (“very unsatisfactory”) to 10 (“completely satisfactory”).

Safety endpoints: Additionally, the compatibility and safety of the IMP was examined by regular documentation of pulse and blood pressure as well as by recording adverse and serious adverse events (AEs and SAEs).

### 2.8. Safety and Compliance

At inclusion, contraindications to beta-blocker treatment were verified through an electrocardiogram and a cardiology consultation [18]. Case report forms (CRF) were used to document and evaluate concomitant illnesses and medications. Any contraindication to participation led to immediate exclusion from the study. The safety parameters pulse and blood pressure were measured regularly throughout the study and documented in patients diaries. Patients were monitored by a physician during the first administration of the nasal spray and for the following four hours. All AEs were documented on an AE form, and seriousness of events and their causal linkage to the medication were assessed by the principal investigator.

In order to ensure the quality of the study, monitoring was carried out at regular intervals according to a pre-specified plan by an independent monitor expert.

To measure compliance, all patients had to document the daily use of the nasal spray in a patient diary. In addition, the nasal spray bottles that were used had to be returned to the study center after use to check the fill level.

### 2.9. Statistical Analyses

Descriptive statistics were used to summarize all data. Normal distributed data are presented using mean and standard deviation (SD) and skewed distributed data using median and 1. to 3. Quartile. Categorical data are presented using absolute and relative frequencies. The primary endpoint (ESS three months after therapy) was analyzed by using a paired t-test comparing both treatment groups. Results are presented as the mean difference accompanied by the 95% confidence interval. To adjust the results for potential carryover and period effects, a linear mixed model was used as a sensitivity analysis. Since the intention-to-treat (ITT) and per protocol (PP) population were identical, only one set of analyses had to be performed.

Secondary endpoints and safety parameters were analyzed in an exploratory manner. For comparison of continuous data, a paired t-test was used for the endpoints ESS, subjective satisfaction and the 5Q-5D-3L. Due to violation of normality assumptions a Wilcoxon signed rank test was used for comparison of HHT-related quality of life (SFB questionnaire item 6) and the blood values hemoglobin, ferritin and transferrin. Differences between means are presented as mean-difference with corresponding 95% confidence intervals, while differences for HHT-related quality of life and blood values were estimated using the median of the differences (cave: this is not equal to the difference of the medians) with the corresponding 95% confidence interval.

The level of significance was set to 5% for the primary endpoint, as well as for all secondary endpoints. Analyses were performed by using the statistical software SAS 9.4 (SAS Institute Inc., Cary, NC, USA).

## 3. Results

### 3.1. Trial Population

In total, 120 patients were assessed for eligibility to participate in the study. Of these, 23 patients met the restrictive inclusion criteria and agreed to participate in the study. However, 3 of the 23 patients were excluded at enrollment based on results of the cardiological examination. Briefly, two patients had bradycardia in the ECG and one patient was suspected to have Brugada syndrome. Thus, 20 HHT patients were randomized. First patient-in was in February 2020 and last patient-in was in June 2021. Accordingly, last patient-out was in September 2021. The baseline characteristics of all participants are shown in Table 1.

**Figure 2:**
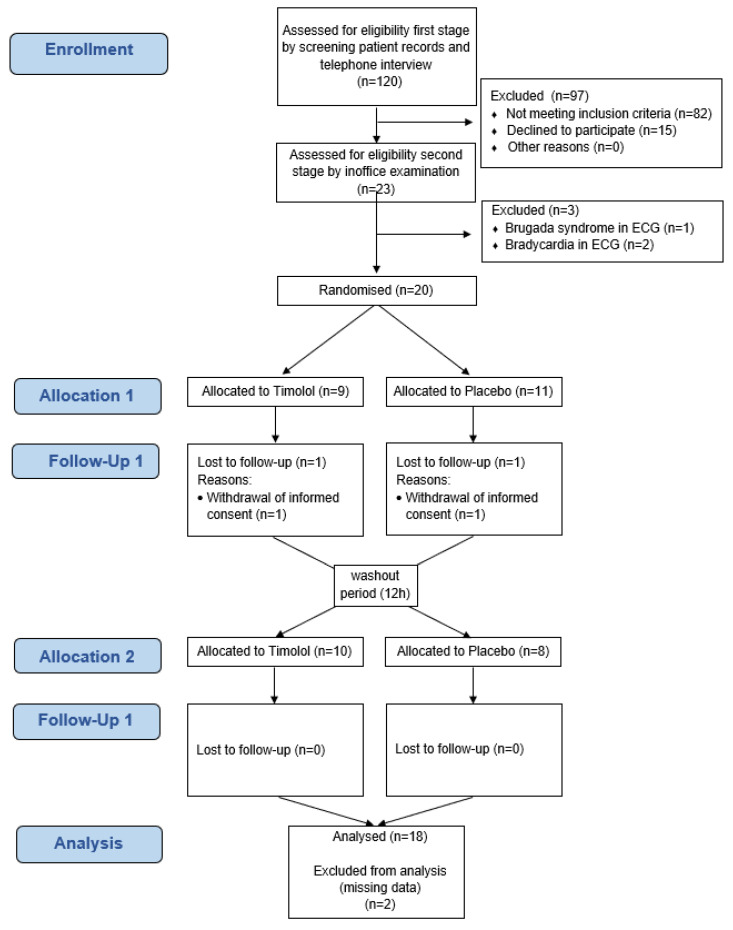
Flow Diagram of patients of the TIM-HHT study.

During the first 3 month period, two patients were lost to follow up through withdrawal of informed consent. One patient of the IMP group stopped treatment after 10 weeks due to increased epistaxis. One more patient of the IMP group experienced personal stress issues and stopped treatment during the 12th week. Both patients had to be excluded from the ITT population, since they provided no information about the primary endpoint. Thus, the ITT population consisted of 18 patients. Since no further major protocol violations were identified, the per-protocol population was identical to the ITT population (see flowchart Figure 2).

### 3.2. Response to Treatment

**Figure 3:**
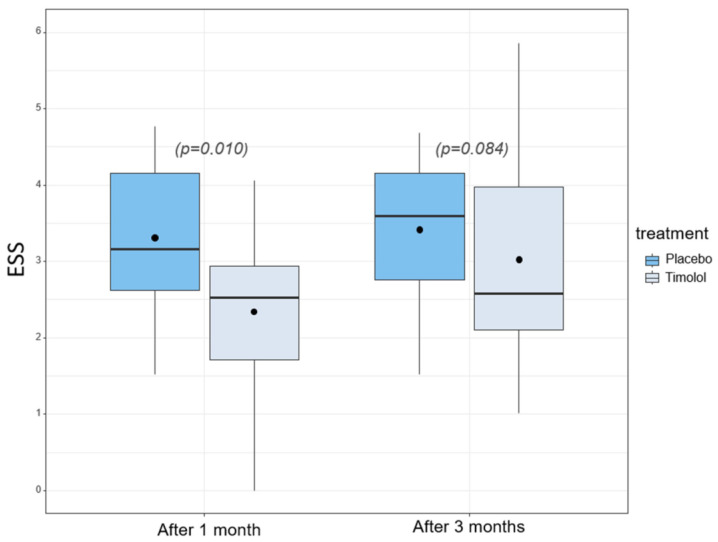
Change in Epistaxis Severity Score after one and three months in the TIM-HHT study.

Primary endpoint: Epistaxis severity after three months failed to reveal significant improvement in the timolol group compared to the placebo group, Δ = 0.39 (95% CI: −0.06, 0.84), *p* = 0.084 (Figure 3). After adjusting for carry-over and period effects (both not significant), the treatment effect slightly improved to Δ = 0.42 (95% CI: −0.01, 0.86), *p* = 0.057, but still fell short of the conventional level of statistical significance (see also Table 2). However, the MID of the ESS (0.71) compared to baseline was reached in the timolol group and was 0.9 in the timolol group compared to 0.5 in the placebo group after three months.

Secondary endpoints: The results of all secondary endpoints are summarized in Table 3. A significant improvement in ESS after 1 month could be seen in the timolol group compared to placebo group (*p* = 0.010). Again, the MID of the ESS (0.71) was obtained in the timolol group (1.6 timolol/0.6 placebo), yet not in the placebo group. The evolution of the subjective satisfaction with epistaxis based on the NRS three months after beginning of treatment showed no significant differences between timolol group and placebo group (*p* = 0.117). Similar to the ESS, the analysis after one month revealed a significant increase in the subjective satisfaction comparing the two treatment groups (*p* = 0.005).

The overall course of the blood values was variable. The development of hemoglobin levels collected throughout the study did not differ between the two study arms (*p* = 0.218). Ferritin levels decreased minimally in both groups, but were significantly higher in the timolol group (*p* = 0.041) after 3 months. Yet, the transferrin value developed slightly positively in both groups, though this development was more pronounced in the placebo group (*p*= 0.039) (see Table 1 and Table 3).

The assessment of quality of life based on the EQ-5D-3L (*p* = 0.863) revealed no difference between the two treatment groups after treatment. At the same time, the evaluation of the HHT-related quality of life (SFB questionnaire item 6) showed significantly higher values in the timolol group (*p* = 0.046).

The safety parameters of blood pressure and pulse as the two most important indicators of systemic side effects were stable over the period of use in all study participants.

### 3.3. Adverse Events

Adverse events that the principal investigator (PI) rated to be “possibly” or “probably” related to the IMP included nasal discomfort (3 timolol group(T)/0 placebo group (P)) and nasal blockage (1T/2P). Further adverse events included drowsiness and dizziness (3T/3P), dyspnea (1T/1P) and headache (2T/1P). Three SAEs in the sense of unscheduled inpatient treatment referrals occurred during the course of the study. The reasons for the hospital stay were due to treatment of AVMs of the GI-tract, tachycardia and epistaxis. The association with the investigational drug was rated as “unlikely” by the investigators.

When comparing the relevant data on adverse events, there was no significant difference between the two study groups regarding intensity of AEs, number of SAEs, frequency of a “possible” or “probable” association with IMP or measures taken in case of adverse events.

## 4. Discussion

This was the first phase 2, prospective, randomized, double−blinded, cross−over trial in which timolol nasal spray was compared to placebo as add−on therapy for epistaxis in HHT patients for a study period of three months. We were able to show a significant effect on the ESS and NRS after one month of use, reaching these secondary endpoints. Although this effect was also visible after 3 months, it flattened out and thus failed to reach the significance level. Therefore, the primary endpoint of the study was not met. Evaluation of further secondary outcomes showed a significant improvement of HHT-related quality of life (SFB questionnaire item 6) compared to the placebo group. The hemoglobin value was stable, as was the evaluation of quality of life using a validated questionnaire (EQ-5D-3L). The evolution of ferritin and transferrin levels was inconsistent overall.

Extensive literature research resulted in two randomized trials that have been published so far on topical treatment with timolol for epistaxis in HHT patients. The first placebo-controlled trial published by Dupuis et al. in 2019 failed to show that topically administered timolol (1 mg/d) used one month was more efficacious than 0.9% saline nasal spray [19]. However, the NOSE study by Whitehead et al. showed an effect of saline in the treatment of epistaxis in HHT patients [20]. It is therefore questionable whether saline is an appropriate placebo. The study by Peterson et al. also failed to demonstrate a relevant difference in the efficacy of timolol compared with placebo [21]. Here, a thermosensitive gel was chosen as placebo, which according to the study itself was highly effective in reducing epistaxis severity.

We chose benzalkonium chloride—the carrier solution of timolol nasal spray—at a dose of 0.01% as the placebo for our study. Benzalkonium chloride has no known positive therapeutical effect on the nasal mucosa. It has the same side effect profile as the IMP carrier solution. In addition, the taste of the drops are comparable, facilitating a true blinding of the participants. We therefore considered it a suitable placebo for our study.

A unique feature of the study at hand is the fact that timolol was applied additively rather than exclusively. This may have contributed to the measurable, positive effect of timolol. It is possible that timolol in particular helps to reduce the recurrence of laser-treated HHT foci. A reason might be that the preceding obliteration of the inflowing and outflowing vessels by means of laser ensures a longer and thus more effective duration of action in these mucosal areas. Furthermore, the drug may penetrate the mucosa more easily when altered by laser treatment. The effect of timolol might flatten out, when the wound healing of endonasal HHT foci is completed.

No serious adverse events related to timolol were seen in this study, and the nasal spray showed good overall tolerability. There was no case of bradycardia or hypotension. Nasal discomfort (3 timolol group(T)/0 placebo group (P)) and nasal blockage (1T/2P) were considered related adverse events most likely being associated with benzalkonium chloride. Nevertheless, pronounced side effects must be taken into account when using timolol intranasally. Based on experiences of administration intraocularly or on the skin, timolol is known to be systemically absorbed with possible cardiovascular effects [18]. Patients with intermediate or poor metabolization of CYP2D6 achieve higher plasma concentrations of timolol which may lead to an increased risk of side effects [9]. Thus it is mandatory to monitor the side effects, such as hypotension and bradycardia, carefully.

A challenge of this study was to find suitable parameters to measure the effect of the IMP compared to placebo. In contrast to individual values describing epistaxis, such as the duration or intensity, the ESS combines six different items to generate a validated score with a defined value for minimal clinical important difference (MID = 0.7). We therefore rated an improvement of the ESS (baseline 3.9 (SD 1.4)) after one month, 2.3 (SD 1.1) for the timolol group and 3.3 (SD 1.1) for the placebo group, as not only statistically significant but as clinically relevant. Furthermore, it was our aim to assess the quality of life of HHT patients. The SFB questionnaire was developed for HHT patients and contains numerous qualitative questions with an embedded numeric, HHT-related quality of life scale (question 6), and showed a statistically significant difference between intervention and control after three months. A recently published NOSE HHT score was not available when designing the present study [22].

Several further limitations of the study have to be discussed. The sample size of this monocenter study was relatively small and the calculated sample size of *n* = 19 was not reached, which may be the reason that the hypothesized effect could not be demonstrated. By choosing a nasal spray instead of drops in the context of our study we intended to reach the anterior segments of the nose as completely as possible, regardless of the location of the HHT foci in this area. Nevertheless, we are aware that the location and type of AV-shunts, which were not evaluated at baseline, could have influenced the extent of efficacy [23]. The chosen blood values were supposed to underline potential changes in epistaxis. However, during the study, participants were neither examined for other possible sources of bleeding (e.g., gastrointestinal, menstruation) nor for infection or liver function. This limits the validity of these parameters. Finally, no direct pharmacokinetic data exist for topical application of timolol, and no measurement of tissue drug levels were taken. Dose determination was based on case reports available at the time of study design and an off−label investigation conducted by the study center [4,8,9].

## 5. Conclusions

The results of this randomized trial provide some evidence that timolol nasal spray can positively affect epistaxis severity and subjective satisfaction with epistaxis in HHT patients when applied additively to standard laser therapy. However, the effect of the drug was observed to diminish over time. Multicenter trials with a larger sample size are warranted to confirm and generalize these findings and to explore more precisely the value of timolol as an additive, local therapy in HHT patients.

## Figures and Tables

**Table 1:** Patients’ characteristics of the ITT population.

Patients’ Characteristics	Value	Study Population (*n* = 18)
Age	Mean (SD), range	49.9 (12.9), 24–76
Gender (male/female)	No. (%)	11 (61%)/7 (39%)
Blood pressure systolic	Mean (SD), range	136 (16), 103–171
Blood pressure diastolic	Mean (SD), range	84 (10), 68–106
Hemoglobin [g/dL]	Median (IQR)	12.1 (10.0, 14.3)
Ferritin [ug/L]	Median (IQR)	39.4 (13.6, 56.8)
Transferrin [mg/dL]	Median (IQR)	294.5 (257.8, 346.3)
ESS baseline	Mean (SD), range	3.9 (1.4), 2.07–6.88
NRS baseline	Mean (SD), range	5.9 (2.4), 0–9
EQ-5D-3L score (utility score)	Mean (SD), range	0.96 (0.06), 0.89–1
EQ-5D-3L VAS	Mean (SD), range	75 (18), 30–95
HHT-related QoL	Median (IQR)	4 (3, 7)

SD = Standard deviation/IQR = Interquartile range.

**Table 2:** Primary endpoint, ESS after 3 months.

Placebo (*n* = 18)	Timolol (*n* = 18)	Method of Analysis	Δ (95% CI)	*p* Value
3.4 (SD 1.0)	3.0 (SD 1.4)	Paired *t*-Test	−0.39 (−0.84, 0.06)	0.084
		Mixed model ^a^	−0.42 (−0.86, 0.01)	0.057

Data show mean (standard deviation); Δ, Difference between both groups; 95% CI, 95% confidence interval; ^a^ considering carry-over and period effects.

**Table 3:** Secondary endpoints.

	Placebo (*n* = 18)	Timolol (*n* = 18)	Δ (95% CI)	*p* Value
1 month assessments				
ESS	3.3 (SD 1.1)	2.3 (SD 1.1)	−0.97 (−1.67, −0.27)	0.010
NRS	5.8 (SD 1.9)	7.5 (SD 1.8)	1.7 (0.6, 2.7)	0.005
3 months assessments				
NRS	5.8 (SD 2.3)	6.8 (SD 2.6)	1.0 (−0.3, 2.3)	0.117
Hemoglobin ^a^ [g/dL]	11.9 (10.3, 14.8)	12.4 (11.5, 14.5)	0.35(−0.25, 1.10) *	0.236
Ferritin [ug/L]	27.2 (11.9, 41.6)	36.9 (23.4, 56.9)	9.9 (0.3, 20.9) *	0.043
Transferrin [mg/dL]	309 (288, 345)	301 (250, 332)	−22 (−37, −1) *	0.039
EQ-5D-3L (utility score)	0.96 (SD 0.05)	0.96 (SD 0.10)	0.00 (−0.06, 0.05)	0.863
EQ-5D-3L (VAS)	72.8 (SD 18.2)	75.2 (SD 17.4)	2.4 (−3.9, 8.6)	0.439
HHT-related QoL	4 (3, 7)	4 (2, 7)	−1 (−2, 0) *	0.046

ESS = Epistaxis Severity Score; NRS = Numerical Rating Scale; VAS = Visual Analogue Scale; HHT = Hereditary Hemorrhagic Telangiectasia; QoL = Quality of Life. Data show mean (standard deviation) or median (q1, q3); Δ (95% CI), difference between both groups with 95% confidence interval. * Values relate to a pseudo median differences with the 95% confidence interval. ^a^ *n* = 17 patients because of a missing value.

## Data Availability

Not applicable.
